# Interactions between Environmental Factors and Melatonin Receptor Type 1A Polymorphism in Relation to Oral Cancer Susceptibility and Clinicopathologic Development

**DOI:** 10.1371/journal.pone.0121677

**Published:** 2015-03-25

**Authors:** Feng-Yan Lin, Chiao-Wen Lin, Shun-Fa Yang, Wei-Jiunn Lee, Yung-Wei Lin, Liang-Ming Lee, Junn-Liang Chang, Wei-Chun Weng, Chien-Huang Lin, Ming-Hsien Chien

**Affiliations:** 1 Institute of Medicine, Chung Shan Medical University, Taichung, Taiwan; 2 Department of Emergency Medicine, Chung Shan Medical University Hospital, Taichung, Taiwan; 3 Institute of Oral Sciences, Chung Shan Medical University, Taichung, Taiwan; 4 Department of Dentistry, Chung Shan Medical University Hospital, Taichung, Taiwan; 5 Department of Medical Research, Chung Shan Medical University Hospital, Taichung, Taiwan; 6 Department of Urology, Wan Fang Hospital, Taipei Medical University, Taipei, Taiwan; 7 Department of Medical Management, Taoyuan Armed Forces General Hospital, Taoyuan County, Taiwan; 8 School of Medicine, Pathology Department, National Defense Medical Center, Taipei, Taiwan; 9 Division of Urology, Department of Surgery, Tungs' Taichung MetroHarbor Hospital, Taichung, Taiwan; 10 Graduate Institute of Medical Sciences, Taipei Medical University, Taipei, Taiwan; 11 Graduate Institute of Clinical Medicine, Taipei Medical University, Taipei, Taiwan; 12 Wan Fang Hospital, Taipei Medical University, Taipei, Taiwan; Tufts University, UNITED STATES

## Abstract

**Background:**

The purpose of this study was to explore the combined effect of melatonin receptor type 1A (*MTNR1A*) gene polymorphisms and exposure to environmental carcinogens on the susceptibility and clinicopathological characteristics of oral cancer.

**Methodology and Principal Findings:**

Three polymorphisms of the *MTNR1A* gene from 618 patients with oral cancer and 560 non-cancer controls were analyzed by real-time polymerase chain reaction (PCR). The CTA haplotype of the studied *MTNR1A* polymorphisms (rs2119882, rs13140012, rs6553010) was related to a higher risk of oral cancer. Moreover, *MTNR1A* gene polymorphisms exhibited synergistic effects of environmental factors (betel quid and tobacco use) on the susceptibility of oral cancer. Finally, oral-cancer patients with betel quid-chewing habit who had T/T allele of *MTNR1A* rs13140012 were at higher risk for developing an advanced clinical stage and lymph node metastasis.

**Conclusion:**

These results support gene-environment interactions of *MTNR1A* polymorphisms with smoking and betel quid-chewing habits possibly altering oral-cancer susceptibility and metastasis.

## Introduction

Oral cavity cancers are among the most common, with an estimated worldwide annual age-standardized incidence of 3.8/100,000 and a mortality rate of 1.9/100,000 persons [1]. The vast majority of these cancers are oral squamous cell carcinomas (OSCCs). Despite substantial efforts and novel therapeutic developments, the 5-year survival rate for OSCC has not appreciably improved over the last 2 decades [2, 3]. In Taiwan, OSCC is also the fourth most common male cancer and the fifth leading cause of cancer death [4]. Therefore, OSCC is still a significant public health threat throughout the world [5].

It is widely accepted that the development of OSCC is a multistep process requiring the accumulation of multiple genetic alterations, which is affected by a patient’s genetic predisposition and by environmental influences, including alcohol and tobacco consumption, betel (*Areca catechu*)-quid chewing, and viral infection [6–8]. Single-nucleotide polymorphisms (SNPs), the most common type of DNA sequence variation, occur when a single nucleotide in the shared sequence of a gene differs between members of a species or paired chromosomes in an individual, and are thought to be associated with the development of certain diseases [9]. According to previous reports, it seems likely that genetic polymorphisms alone are unable to elicit clinical manifestations of OSCC, but together with lifestyle and environmental factors, they might further contribute to the development and progression of the disease [10, 11].

Melatonin is a hormone produced by the pineal gland and is released in response to photic information from the retina. In humans, melatonin secretion increases soon after exposure to darkness, peaks during the middle of the night, and then decreases over the second half of the night [12]. Melatonin was reported to exert oncostatic activity through biological mechanisms including antiproliferative and proapoptotic actions, stimulation of anticancer immunity, modulation of oncogene expression, and anti-inflammatory, antioxidant, and antiangiogenic effects [13, 14]. The anticancer effects of melatonin were indicated in a wide range of different tumors (breast, gastrointestinal, hematological, prostate, osteosarcoma, and melanoma) [14]. However, little research has been conducted into melatonin and its anticancer activity in the oral cavity.

The melatonin receptors 1A (MTNR1A) and 1B (MTNR1B) are largely responsible for mediating the downstream effects of melatonin, while arylalkylamine N-acetyltransferase (AANAT) is the major enzyme in melatonin synthesis, and controls the day/night rhythm of melatonin production by the pineal gland [15]. All three have been identified as potentially important players in mediating breast cancer risk [16, 17], but only MTNR1A was reportedly correlated with tumor sizes and survival rates in patients with OSCC [18]. Studies demonstrated that polymorphisms in *MNTRs* are associated with several kinds of disease including rheumatoid arthritis [19], breast cancer [20], acute myocardial infarction [21], calcium nephrolithiasis [22], and polycystic ovary syndrome (PCOS) [23], suggesting functional roles for these variants. It was suggested as possibly being due to altered protein production or function.

**Fig 1:**
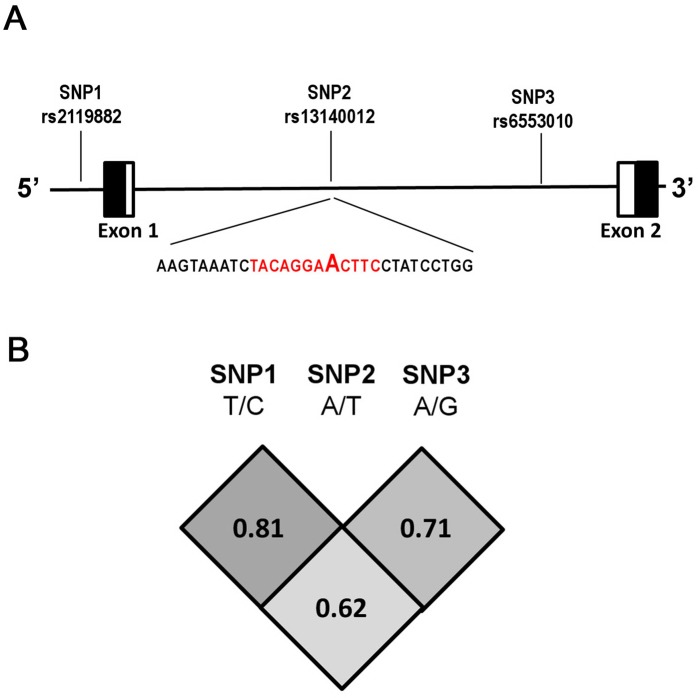
Melatonin receptor (*MTNR*) 1A gene, locations of the genotyped variants, and their pairwise linkage disequilibrium (LD) patterns. Schematic presentation of the *MTNR1A* (gene ID: 4543) (A) indicating locations of the analyzed variants (rs2119882, rs13140012, and rs6553010), (B) the one observed haploblock, and the pairwise LD measure, D’. Black box, untranslated region; white box, coding region. The red color reveals the putative transcription factor binding sites.

Although evidence exists supporting MTNR1A having a tumor-suppressive effect, little is known about the association between genetic polymorphisms of *MTNR1A* and the risk of oral cancer. The current study investigated relationships between SNPs (rs2119882) in the promoter and intron (rs13140012 and rs6553010) regions of the *MTNR1A* gene and the risk of oral cancer (Fig. 1A). The influences of these SNPs combined with betel-nut chewing and tobacco consumption, leading to a susceptibility to oral cancer, were evaluated. We also investigated relationships among genetic influences, environmental exposures, and clinicopathological characteristics of oral cancer. To our knowledge, this is the first study to demonstrate a significant association between *MTNR1A* polymorphisms and oral carcinogenesis.

## Materials and Methods

### Subjects and specimen collection

In 2007–2013, we recruited 618 patients (596 males and 22 females with a mean age of 54.29 ± 11.28 years) at Chung Shan Medical University Hospital, Taichung, and Changhua Christian Hospital and Show Chwan Memorial Hospital, Changhua, Taiwan as the case group. Meanwhile, controls were enrolled from the physical examination during those three hospitals, which are also the facilities that cases were collected from. At the end of recruitment, a total of 560 participants (457 males and 103 females with a mean age of 51.82 ± 14.72 years) that had neither self-reported history of cancer of any sites were included. In addition, subjects with oral precancerous disease such as oral submucous fibrosis, leukoplakia, erythroplakia, verrucous hyperplasia, etc. were excluded from control group. The participation rate was approximately 92.9% (618/665) for cases and 80.8% (560/693) for controls. For both cases and controls, we used a questionnaire to obtain exposure information about betel-quid chewing, tobacco use, and alcohol consumption. Medical information of the cases, including TNM clinical staging, the primary tumor size, lymph node involvement, and histologic grade, was obtained from their medical records. Oral-cancer patients were clinically staged at the time of their diagnosis according to the TNM staging system of the American Joint Committee on Cancer (AJCC) Staging Manual (7^th^ ed.): stage I = T1N0M0; stage II = T2N0M0; stage III = T3N0M0, or T1, T2, or T3N1M0; and stage IV = any T4 lesion, any N2 or N3 lesion, or any M1 lesion. Tumor differentiation was examined by a pathologist according to the AJCC classification. Whole-blood specimens collected from controls and OSCC patients were placed in tubes containing ethylenediaminetetraacetic acid (EDTA), immediately centrifuged, and then stored at -80°C. This study was approved by the Institutional Review Boards of Chung Shan Medical University Hospital, and informed written consent to participate in the study was obtained from each individual.

### Selection of *MTNR1A* polymorphisms

In total, 3 SNPs in *MTNR1A* were selected from the International HapMap Project data for this study. We included -386A/G (rs2119882) in the promoter region. rs13140012 and rs6553010, which are located in the intron 1 of *MTNR1A*, were selected in this study since these 2 SNPs were found to modify the binding affinities of several transcription factors [22].

### Genomic DNA extraction

Genomic DNA was extracted using QIAamp DNA blood mini kits (Qiagen, Valencia, CA, USA) following the manufacturer’s instructions. We dissolved DNA in TE buffer (10 mM Tris and 1 mM EDTA; pH 7.8) and then quantified it by measuring the optical density at 260 nm. The final preparation was stored at -20°C and used to create templates for the polymerase chain reaction (PCR).

### Real-time PCR

Allelic discrimination of rs2119882, rs13140012, and rs6553010 polymorphisms of the *MTNR1A* gene was assessed with the ABI StepOne Real-Time PCR System (Applied Biosystems, Foster City, CA, USA), and analyzed with SDS vers. 3.0 software (Applied Biosystems) using a TaqMan assay. The final volume of each reaction was 5 μL, containing 2.5 μL TaqMan Genotyping Master Mix, 0.125 μL TaqMan probe mix, and 10 ng genomic DNA. The real-time PCR included an initial denaturation step at 95°C for 10 min, followed by 40 cycles at of 95°C for 15 s and then at 60°C for 1 min.

### Statistical analysis

Differences between the 2 groups were considered significant for *p* values of < 0.05. Hardy-Weinberg equilibrium (HWE) was assessed using a goodness-of-fit *Χ* ^*2*^-test for biallelic markers. The Mann-Whitney *U*-test and Fisher’s exact test were used to compare differences in distributions of patient demographic characteristics between the non-cancer (control) and oral-cancer groups. The adjusted odds ratios (ORs, AORs) and 95% confidence intervals (CIs) of the association between genotype frequencies and risk plus clinicopathological characteristics were estimated using multiple logistic regression models, after controlling for other covariates. We analyzed all data with Statistical Analytic System (SAS Institute, Cary, NC, USA) software for Windows.

## Results

The statistical analysis of demographic characteristics is shown in Table 1. We found significantly different distributions of age (*p* = 0.001), gender (*p* < 0.001), betel-quid chewing (*p* < 0.001), alcohol consumption (*p* < 0.001), and tobacco use (*p* < 0.001) between control participants and OSCC patients. To diminish the possible interference of environmental factors, the AORs with 95% CIs were estimated by multiple logistic regression models after controlling for other covariates in each comparison.

**Table 1:** Distributions of demographical characteristics in 560 controls and 618 patients with oral cancer.

**Variable**	**Controls (*N* = 560)**	**Patients (*N* = 618)**	***p* value**
**Age (years)**	**Mean ± S.D.**	**Mean ± S.D.**	
	51.82 ± 14.72	54.29 ± 11.28	0.001*
**Gender**	***n* (%)**	***n* (%)**	
Male	457 (81.6%)	596 (96.4%)	
Female	103 (18.4%)	22 (3.6%)	<0.001*
**Betel nut chewing**			
No	467 (83.4%)	140 (22.7%)	
Yes	93 (16.6%)	478 (77.3%)	<0.001*
**Tobacco consumption**			
No	340 (60.7%)	90 (14.6%)	
Yes	220 (39.3%)	528 (85.4%)	<0.001*
**Alcohol consumption**			
No	346 (61.8%)	260 (42.1%)	
Yes	214 (38.2%)	358 (57.9%)	<0.001*
**Clinical stage**			
Stage I/II		277 (44.8%)	
Stage III/IV		341 (55.2%)	
**Tumor size**			
≤ T2		379 (61.3%)	
> T2		239 (38.7%)	
**Lymph node metastasis**			
No		399 (64.6%)	
Yes		219 (35.4%)	
**Distant metastasis**			
No		610 (98.7%)	
Yes		8 (1.3%)	
**Cell differentiation**			
Well		83 (13.4%)	
Moderately or poorly		535 (86.6%)	

* *p* < 0.05, statistically significant.

In our recruited control group, frequencies of *MTNR1A* genes were in Hardy-Weinberg equilibrium (*p* > 0.05). Reconstructed linkage disequilibrium (LD) plots for the 3 SNPs are shown in Fig. 1B. The genotype distributions and associations between oral cancer and gene polymorphisms of *MTNR1A* are shown in Table 2. Alleles with the highest distribution frequencies for rs2119882, rs13140012, and rs6553010 genotys of *MTNR1A* in both recruited oral cancer patients and healthy controls were heterozygous T/C, heterozygous A/T, and homozygous A/A, respectively. After adjusting for the variables, there was no significant difference in having oral cancer in individuals with the rs2119882, rs13140012, and rs6553010 polymorphisms of the *MTNR1A* gene compared to wild-type (WT) individuals.

**Table 2:** Distribution frequencies of *MTNR1A* genotypes in 560 controls and 618 oral-cancer patients.

**Variable**	**Controls (*N* = 560) *n* (%)**	**Patients (*N* = 618) *n* (%)**	**OR (95% CI)**	**AOR (95% CI)**
**rs2119882**				
TT	232 (41.4%)	234 (37.9%)	1.00	1.00
TC	263 (47.0%)	297 (48.1%)	1.120 (0.875~1.432)	0.857 (0.602~1.222)
CC	65 (11.6%)	87 (14.0%)	1.327 (0.917~1.919)	1.331 (0.766~2.313)
TC+CC	328 (58.6%)	384 (62.1%)	1.161 (0.919~1.467)	0.933 (0.665~1.308)
**rs2119882 Alleles**	**Controls (*N* = 1120) *n* (%)**	**Patients (*N* = 1236) *n* (%)**		
T	727 (64.9%)	765 (61.9%)	1.00	
C	393 (35.1%)	471 (38.1%)	1.139 (0.963~1.347)	———-
**rs13140012**				
AA	214 (38.2%)	251 (40.6%)	1.00	1.00
AT	270 (48.2%)	288 (46.6%)	0.909 (0.711~1.164)	0.870 (0.609~1.243)
TT	76 (13.6%)	79 (12.8%)	0.886 (0.616~1.275)	1.045 (0.611~1.788)
AT+TT	346 (61.8%)	367 (59.4%)	0.904 (0.716~1.143)	0.904 (0.644~1.269)
**rs13140012 Alleles**	**Controls (*N* = 1120) *n* (%)**	**Patients (*N* = 1236) *n* (%)**		
A	698 (62.3%)	790 (63.9%)	1.00	
T	422 (37.7%)	446 (36.1%)	0.934 (0.790~1.104)	———-
**rs6553010**				
AA	240 (42.9%)	275 (44.5%)	1.00	1.00
AG	238 (42.5%)	265 (42.9%)	0.972 (0.760~1.243)	0.809 (0.566~1.157)
GG	82 (14.6%)	78 (12.6%)	0.830 (0.582~1.184)	0.711 (0.426~1.186)
AG+GG	320 (57.1%)	343 (55.5%)	0.935 (0.743~1.178)	0.783 (0.561~1.094)
**rs6553010 Alleles**	**Controls (*N* = 1120) *n* (%)**	**Patients (*N* = 1236) *n* (%)**		
A	718 (64.1%)	815 (65.9%)	1.00	
G	402 (35.9%)	421 (34.1%)	0.923 (0.779~1.093)	———-

Odds ratios (ORs) and with their 95% confidence intervals (CIs) were estimated by logistic regression models. Adjusted ORs (AORs) with their 95% CIs were estimated by multiple logistic regression models after controlling for age, gender, betel nut chewing, and tobacco and alcohol consumption.

Combined effects of environmental factors and *MTNR1A* gene SNPs on the risk of oral cancer are shown in Tables 3 and 4. Among 748 smokers, subjects with at least 1 C allele of rs2119882, 1 T allele of rs13140012, or 1 G allele of rs6553010 and the betel-nut-chewing habit respectively had 42.00- (95% CI: 15.79~111.71), 27.96- (95% CI: 11.03~70.84), and 23.65-fold (95% CI: 10.20~54.86) higher risks of having oral cancer. Individuals with either at least 1 C allele of rs2119882, 1 T allele of rs13140012, or 1 G allele of rs6553010 or who chewed betel nut had respective risks of 5.17- (95% CI: 2.75~9.70), 4.39- (95% CI: 2.30~8.36), and 4.15-fold (95% CI: 2.32~7.42) of having oral cancer compared to individuals with WT homozygotes who did not chew betel nut (Table 3).

**Table 3:** Adjusted odds ratios (ORs, AORs) and 95% confidence intervals (CIs) of oral cancer associated with *MTNR1A* genotypic frequencies and betel nut chewing among 748 smokers.

**Variable**	**Controls (*N* = 220) (%)**	**Patients (*N* = 528) (%)**	**OR (95% CI)**	**AOR (95% CI)**
**rs2119882**				
^a^ TT genotype & non-betel nut chewing	60 (27.3%)	29 (5.5%)	1.00	1.00
^b^ TC or CC genotype or betel nut chewing	112 (50.9%)	213 (40.3%)	**3.935 (2.389~6.480)**	**5.168 (2.754~9.700)**
^c^ TC or CC genotype with betel nut chewing	48 (21.8%)	286 (54.2%)	**12.328 (7.195~21.122)**	**41.998 (15.790~111.707)**
**rs13140012**				
^a^ AA genotype & non-betel nut chewing	53 (24.1%)	32 (6.1%)	1.00	1.00
^b^ AT or TT genotype or betel nut chewing	124 (56.4%)	227 (43.0%)	**3.032 (1.857~4.951)**	**4.388 (2.304~8.357)**
^c^ AT or TT genotype with betel nut chewing	43 (19.5%)	269 (50.9%)	**10.361 (6.013~17.855)**	**27.958 (11.034~70.842)**
**rs6553010**				
^a^ AA genotype & non-betel nut chewing	57 (25.9%)	36 (6.8%)	1.00	1.00
^b^ AG or GG genotype or betel nut chewing	122 (55.5%)	236 (44.7%)	**3.063 (1.912~4.906)**	**4.151 (2.321~7.424)**
^c^ AG or GG genotype with betel nut chewing	41 (18.6%)	256 (48.5%)	**9.886 (5.809~16.826)**	**23.652 (10.197~54.862)**

ORs with their 95% CIs were estimated by logistic regression models.

AORs with their 95% CIs were estimated by multiple logistic regression models after controlling for age, gender, and alcohol consumption.

^a^ Individuals with a wild genotype but without betel nut chewing.

^b^ Individuals with either at least one mutated genotype or betel nut chewing.

^c^ Individuals with at least one mutated genotype and betel nut chewing.

**Table 4:** Adjusted odds ratios (ORs, AORs) and 95% confidence interval (CIs) of oral cancer associated with *MTNR1A* genotypic frequencies and smokers among 571 betel nut consumers.

**Variable**	**Controls (*N* = 93) (%)**	**Patients (*N* = 478) (%)**	**OR (95% CI)**	**AOR (95% CI)**
**rs2119882**				
^a^ TT genotype & non-smoker	10 (10.8%)	11 (2.3%)	1.00	1.00
^b^ TC or CC genotype or smoker	35 (37.6%)	181 (37.9%)	**4.701 (1.855~11.912)**	**12.954 (3.073~-54.599)**
^c^ TC or CC genotype with smoking	48 (51.6%)	286 (59.8%)	**5.417 (2.182~13.447)**	**9.475 (2.580~34.793)**
**rs13140012**				
^a^ AA genotype & non-smoker	6 (6.5%)	11 (2.3%)	1.00	1.00
^b^ AT or TT genotype or smoker	44 (47.3%)	198 (41.4%)	2.455 (0.862~6.993)	4.230 (0.723~24.761)
^c^ AT or TT genotype with smoking	43 (46.2%)	269 (56.3%)	**3.412 (1.199~9.707)**	**8.369 (1.843~38.005)**
**rs6553010**				
^a^ AA genotype & non-smoker	5 (5.4%)	11 (2.3%)	1.00	1.00
^b^ AG or GG genotype or smoker	47 (50.5%)	211 (44.1%)	2.041 (0.677~6.151)	4.285 (0.811~22.633)
^c^ AG or GG genotype with smoking	41 (44.1%)	256 (53.6%)	2.838 (0.938~8.589)	**5.329 (1.170~24.277)**

ORs with their 95% CIs were estimated by logistic regression models.

AORs with their 95% CIs were estimated by multiple logistic regression models after controlling for age, gender, and alcohol consumption.

^a^ Individuals with the wild-type genotype who did not smoke.

^b^ Individuals with either at least one mutated genotype or who smoked.

^c^ Individuals with both at least one mutated genotype and who smoked.

Among betel-nut consumers in our cohort, subjects with *MTNR1A* polymorphic rs2119882, rs13140012, or rs6553010 genes and who smoked had corresponding 9.48- (95% CI: 2.58~34.79), 8.37- (95% CI: 1.84~38.01), and 5.33-fold (95% CI: 1.17~22.28) higher risks of having oral cancer compared to betel-quid chewers with the WT gene who did not smoke (Table 4). Moreover, people who were either polymorphic for *MTNR1A* in rs2119882 or who smoked were at a 12.95-fold risk (*p* < 0.05) of developing oral cancer, compared to people with the WT gene who did not smoke (Table 4). The above results suggest that *MTNR1A* gene polymorphisms have strong impacts on oral-cancer susceptibility in betel-nut chewers and/or cigarette smokers. About evaluating the interactions between *MTNR1A* SNPs and betel nut chewing/smoking among non-smokers/non-chewers cohort. Because the sample size of non-smokers (90 cases) or non-chewers (140 cases) in our recruited OSCC patients are relative too small to further divide into 3 subgroups (WT alleles, heterozygous mutant genotype and homozygous mutant genotype). We suggested that the interactions between *MTNR1A* SNPs and betel nut chewing/smoking among non-smokers/non-chewers could not be evaluated right now and more samples should be collected in our future work.

To explore the effects of polymorphic genotypes of *MTNR1A* on the clinical status of OSCC, we classified OSCC patients into 3 subgroups. In the first subgroup, patients had homozygous WT alleles; in the other 2 subgroups they had 1 polymorphic allele and 2 polymorphic alleles, respectively. No significant associations of the rs2119882, rs13140012, and rs6553010 gene polymorphisms with the clinicopathologic status were observed. However, among 478 oral-cancer patients who chewed betel quid, those that had a polymorphic rs13140012 (T/T) gene had a higher risk of developing an advanced clinical stage (AOR: 2.76; 95% CI: 1.27~5.99; *p* = 0.01) and neck lymph node metastasis (AOR: 2.19; 95% CI: 1.01~4.74; *p* = 0.046) compared to patients with the rs13140012 WT, but there were no differences in the primary tumor size, distal metastasis, or histologic grade (Table 5).

**Table 5:** Adjusted odds ratios (ORs, AORs) and 95% confidence intervals (CIs) of clinical statuses associated with genotypic frequencies of MTNR1A rs13140012 in oral cancer among 478 betel quid chewers.

**Clinical stage**				
***MTNR1A* rs13140012**	Stage I+II (*N* = 213) (%)	Stage III+IV (*N* = 265) (%)	AOR (95% CI)	*p* value
**AA**	93 (43.7%)	100 (37.8%)	1.00	
**AT**	101 (47.4%)	126 (47.5%)	1.372 (0.868~2.168)	0.176
**TT**	19 (8.9%)	39 (14.7%)	**2.759 (1.270~5.994)** *	**0.010** *
**Tumor size**				
**MTNR1A rs13140012**	≤ T2 (*N* = 292) (%)	> T2 (*N* = 186) (%)	AOR (95% CI)	*p* value
**AA**	121 (41.5%)	72 (38.7%)	1.00	
**AT**	140 (47.9%)	87 (46.8%)	1.107 (0.696~1.762)	0.667
**TT**	31 (10.6%)	27 (14.5%)	1.311 (0.610~2.814)	0.488
**Lymph node metastasis**				
**MTNR1A rs13140012**	No (*N* = 311) (%)	Yes (*N* = 167) (%)	AOR (95% CI)	*p* value
**AA**	132 (42.4%)	61 (36.5%)	1.00	
**AT**	145 (46.6%)	82 (49.1%)	**1.809 (1.115~2.934)**	**0.016** *
**TT**	34 (10.9%)	24 (14.4%)	**2.190 (1.013~4.736)** *	**0.046** *
**Distant metastasis**				
**MTNR1A rs13140012**	No (*N* = 474) (%)	Yes (*N* = 4) (%)	AOR (95% CI)	*p* value
**AA**	190 (40.1%)	3 (75.0%)	1.00	
**AT**	227 (47.9%)	0 (0%)	——-	
**TT**	57 (12.0%)	1 (25.0%)	1.126 (0.154~12.562)	0.926
**Cell differentiated grade**				
**MTNR1A rs13140012**	≦Grade I (*N* = 65) (%)	>Grade I (*N* = 413) (%)	AOR (95% CI)	*p* value
**AA**	22 (33.8%)	171 (41.4%)	1.00	
**AT**	38 (58.5%)	189 (45.8%)	0.627 (0.326~1.206)	0.162
**TT**	5 (7.7%)	53 (12.8%)	1.720 (0.518~5.713)	0.376

AORs with their 95% CIs were estimated by multiple logistic regression models, after controlling for gender, age, and alcohol and tobacco consumption.

> T2: multiple tumors more than 2 cm.

Cell differentiated grade: grade I: well differentiated; grade II: moderately differentiated; grade III: poorly differentiated.

* *p* < 0.05, statistically significant.

We further explored the haplotypes to evaluate the combined effects of the 3 polymorphisms on oral-cancer susceptibility. The distribution frequencies of the *MTNR1A* rs2119882, rs13140012, and rs6553010 haplotypes in our recruited individuals were analyzed. The most common haplotype in the control was TAA (56.2%), and it was therefore chosen as a reference. Compared to the reference, 1 *MTNR1A* haplotype, CTA, significantly (*p* = 0.001) increased the risks of OSCC by 1.77-fold (95% CI: 1.27~2.47) (Table 6).

**Table 6:** Distribution frequencies of the *MTNR1A* haplotype in controls and oral cancer patients.

**Variable**			**Controls (*N* = 1120) *n* (%)**	**Patients (*N* = 1236) *n* (%)**	**OR (95% CI)**			***p* value**
**rs2119882 T/C**	**rs13140012A/T**	**rs6553010 A/G**						
T	A	A	629 (56.2%)	674 (54.5%)		Reference		
C	T	G	296 (26.4%)	299 (24.2%)		0.943 (0.776~1.145)	0.551	
C	T	A	60 (5.3%)	114 (9.2%)		**1.773 (1.274~2.469)**	0.001*	
Others#			135 (12.1%)	149 (12.1%)		1.030 (0.797~1.332)	0.822	

#Others: TTG(92), TAG (90),CAA (49), CAG (46), TTA (7).

OR, odds ratio; CI, confidence interval.

## Discussion

In this study, we provide novel information of *MTNR1A* SNPs with oral cancer susceptibility, interactions with environmental risk factors, and associations with clinicopathologic statuses.

The pineal hormone, melatonin, most widely recognized for its role in sleep and circadian rhythm regulation, was demonstrated to exert oncostatic effects both *in vivo* and *in vitro* in various types of malignancies, such as cancers of the breast and prostate, and gliomas [24–26]. In mammals, various binding sites for melatonin have been identified and the membrane receptors MTNR1A and MTNR1B, which are of utmost chronobiological importance. *MTNR1B* gene variants were reported to be a risk factor for developing type 2 diabetes [27]. MTNR1A is vastly more abundant than MTNR1B, and there is evidence that melatonin’s growth-inhibitory effects on several cancer cells are MTNR1A receptor-dependent [28, 29]. In addition to receptor-dependent anticancer effects, melatonin has been reported to traverse membranes easily and exert several receptor-independent anticancer effects. For example, melatonin can induce receptor-independent anticancer effects through its interactions with CaM, the PI3K/Akt/ ERK pathway, modulating Sirt1, the ROS balance, and even activation/inhibition of caspases and other proapoptotic (Bam, Bax, Bak) or anti-apoptotic proteins (Bcl-xl, Bcl-2) [14]. In OSCC, melatonin and MTNR1A were reported to exhibit growth-suppressive activity and found that the *MTNR1A* gene is usually downregulated or silenced through epigenetic regulation [18, 30, 31]; however, there have been no studies on the relationship between genetic regulation of the *MTNR1A* gene and oral cancer.

The *MTNR1A* gene is located on chromosome 4q35.1, and is composed of 2 exons that encode a protein of 350 amino acids. A previous study showed that an aberrant variant in the promoter region of *MTNR1A* was inversely correlated with its expression in OSCC lines [18]. Furthermore, no mutation was detected in any of the coding exons (exons 1 and 2) of the *MTNR1A* gene in any of the cell lines tested, which proved that the promoter of *MTNR1A* is a functional region and is associated with MTNR1A expression [18]. SNP rs2119882 is located in the promoter region of the *MTNR1A* gene. According to HapMap [32], rs2119882 can capture the other 2 SNPs (rs11721818 and rs7687823) in the promoter region of the *MTNR1A* gene. The 3 SNPs covered 6.3 kb of the promoter region of the *MTNR1A* gene. Therefore, rs2119882 is the main functional SNP in *MTNR1A*. In addition, previous reports also indicated that a fragment containing exon 1 and intron 1 within the *MTNR1A* gene showed remarkable transcriptional activity [18], and polymorphisms of rs13140012 can affect the binding affinities of several transcription factors [22]. According to those results, SNP rs2119882 and 2 SNPs (rs13140012 and rs6553010) located in intron 1 of *MTNR1A* were investigated in our study.

In our study, *MTNR1A* gene SNPs (rs2119882, rs13140012, and rs6553010) alone did not contribute to oral-cancer susceptibility. The synergistic effects of environmental factors (betel quid chewing and cigarette smoking) and *MTNR1A* gene polymorphisms on the risk of oral cancer are well demonstrated. Similar to our study, our previous studies showed that genetic polymorphisms of an oncogene (e.g., *CA-9*) or tumor suppressor gene alone (e.g., *RECK*) were unable to predict the risk of oral cancer. However, after combined with information on carcinogen exposure, a significant effect for predicting oral-cancer susceptibility was observed [10, 11]. Carcinogen exposure and a possible genetic predisposition may vary between different geographic areas. A Taiwanese cohort study [33] showed that betel quid chewing and smoking habits were risk factors for developing oral cancer. In this study, higher ratios of individuals with betel quid chewing and smoking habits among oral-cancer patients (77.3% and 85.4%) than in the controls (16.6% and 39.3%) were found, which indicates that betel quid chewing and tobacco smoking habits are highly associated with increased risks of oral cancer. Moreover, the synergistic effect of betel quid chewing and smoking in developing oral cancer can be explained by some previous studies. The betel quid used in Taiwan contains areca nut, lime, and piper betel inflorescence or leaf [34]. Hydroxychavicol, a phenolic component of betel leaf, has the capacity to modulate the toxic effects mediated by the cigarette carcinogen, benzo[a]pyrene, by inducing *dihydrodiol dehydrogenase* gene mutations [35]. Evidence showed that alkaline saliva generated by chewing betel quid may play a role in cigarette-related nicotine-induced DNA damage, and reactive oxygen species may be involved in generating this DNA damage [36]. At present, we know that ectopic expression of MTNR1A can suppress the growth of OSCC cells, but the expression of MTNR1A in OSCC is usually downregulated compared to normal oral epithelial cells [18]. Previous reports indicated that betel-quid and tobacco carcinogens can induce expression of the hypoxia-inducible transcription factor, early growth response gene 1 (Egr-1), in buccal fibroblasts [37] and lung tissues [38], respectively. Egr-1 is recognized as a transcriptional suppressor of the *MTNR1A* gene [39]. Moreover, the rs2119882 SNP was also reported to result in quantitative changes of *MTNR1A* in patients with PCOS [23]. However, we have no evidence that polymorphisms of rs2119882, rs13140012, or rs6553010 can directly influence *MTNR1A* expression in patients with OSCC. According to our present data, we hypothesized that betel-quid and tobacco carcinogens might alter *MTNR1A* promoter activity dependent on the presence of rs2119882, rs13140012, and rs6553010 polymorphisms, but this issue should be further investigated in the future.

It was reported that *MTNR1A* in OSCC is usually shown to be diminished and *MTNR1A* promoter methylation has a higher incidence in OSCC compared to corresponding normal mucosa. The protein expression of MTNR1A in OSCC was inversely associated with the T stage and overall survival [18]. In the present study, betel quid-chewing oral-cancer patients with the *MTNR1A* rs13140012 T/T mutation type had higher risks for developing advanced clinical stage and lymph node metastasis than those with the WT. This result also implies an affinity of betel-quid carcinogens in MTNR1A's function and its expression, and then oral cancer more easily proceeds to an advanced stage and metastasis. Several amino acid mutations in MTNR1A have been reported to influence the binding affinity of melatonin [40], but the SNPs we investigated here all located on promoter or intron region and cannot induce the amino acid change on MTNR1A. Moreover, the MTNR1A rs13140012 A>T mutation has been reported to affect the binding affinity of several transcription factor(s) [22], and we suggested that the rs13140012 A>T variant may act in combination with betel-quid carcinogens and other yet to be identified functional variants in the gene to influence MTNR1A expression and the risks for developing advanced clinical stage and metastasis in OSCC patients. However, the underlying mechanism should be elucidated in the laboratory and clinically.

A variety of SNPs may be silent, that is to say, with no direct effect on gene products. However, by virtue of LD that exists across the human genome, they can still be used as genetic markers to locate adjacent functional variants that contribute to disease. When each SNP constructing haplotype has a true contribution to the susceptibility of disease, even though unapparent, haplotype analyses can provide a greater statistical power and are sometimes advantageous over analysis of an individual SNP for detecting an association between alleles and a disease phenotype [41]. We analyzed contributions of different haplotype combinations of 3 *MTNR1A* SNPs (rs2119882, rs13140012, and rs6553010) to the risk of oral cancer and eventually found that the CTA haplotype showed a high risk for OSCC. It is possible that the CTA haplotype of *MTNR1A* is in LD with other functional polymorphisms that are responsible for a susceptibility to OSCC.

In summary, to our knowledge, this is the first study to show the statistical association among the *MTNR1A* polymorphism, betel quid-chewing and tobacco smoking habits, and the susceptibility to oral cancer development. Betel quid-chewing oral-cancer patients with the *MTNR1A* rs13140012 T/T polymorphism had a higher risk of developing an advanced clinical stage and neck lymph node metastasis than WT carriers. However, we are still lacking a compelling mechanistic explanation for this phenomenon and should be further investigated in the future.
